# Gelling without Structuring: A SAXS Study of the Interactions among DNA Nanostars

**DOI:** 10.1021/acs.langmuir.0c01520

**Published:** 2020-08-10

**Authors:** Francesco Spinozzi, Maria Grazia Ortore, Giovanni Nava, Francesca Bomboi, Federica Carducci, Heinz Amenitsch, Tommaso Bellini, Francesco Sciortino, Paolo Mariani

**Affiliations:** † Department of Life and Environmental Sciences, Polytechnic University of Marche, 60131 Ancona, Italy; ‡ Department of Medical Biotechnology and Translational Medicine, Università degli Studi di Milano, 20133 Milan, Italy; ¶ Department of Physics, Sapienza, Università di Roma, 00185 Rome, Italy; § Institute for Inorganic Chemistry, Graz University of Technology, 8010 Graz, Austria

## Abstract

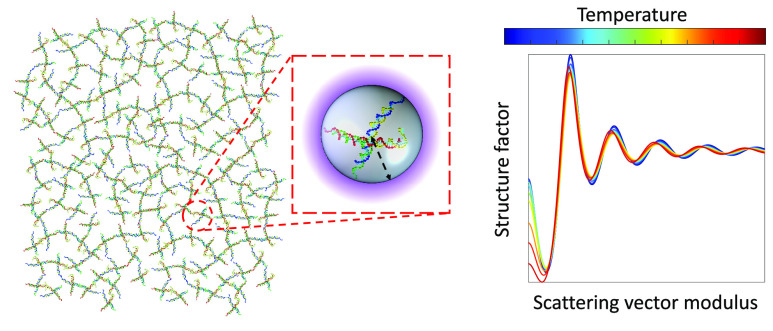

We evaluate, by means of synchrotron small-angle X-ray scattering, the shape and mutual interactions of DNA tetravalent nanostars as a function of temperature in both the gas-like state and across the gel transition. To this end, we calculate the form factor from coarse-grained molecular dynamics simulations with a novel method that includes hydration effects; we approximate the radial interaction of DNA nanostars as a hard-sphere potential complemented by a repulsive and an attractive Yukawa term; and we predict the structure factors by exploiting the perturbative random phase approximation of the Percus–Yevick equation. Our approach enables us to fit all the data by selecting the particle radius and the width and amplitude of the attractive potential as free parameters. We determine the evolution of the structure factor across gelation and detect subtle changes of the effective interparticle interactions, that we associate to the temperature and concentration dependence of the particle size. Despite the approximations, the approach here adopted offers new detailed insights into the structure and interparticle interactions of this fascinating system.

## Introduction

In the last decades, DNA has acquired an increasing importance in material science applications.^1^ Exploiting the addressability of the base-pairing, DNA nanoparticles, and DNA origami of complex shape and binding ability have been designed and realized in the laboratory, providing an alphabet of bricks which can be properly combined to assemble materials with desired properties. By multistep self-assembly of DNA oligomers, cubes, tetrahedra,^2^ octahedra,^3^ tiles,^4^ nanostars with different arms, tubes,^5^ and many other complex shapes have been created. Significantly more complex objects have resulted with the DNA-origami method^6^ by folding and stapling long DNA sequences.

**Figure 1:**
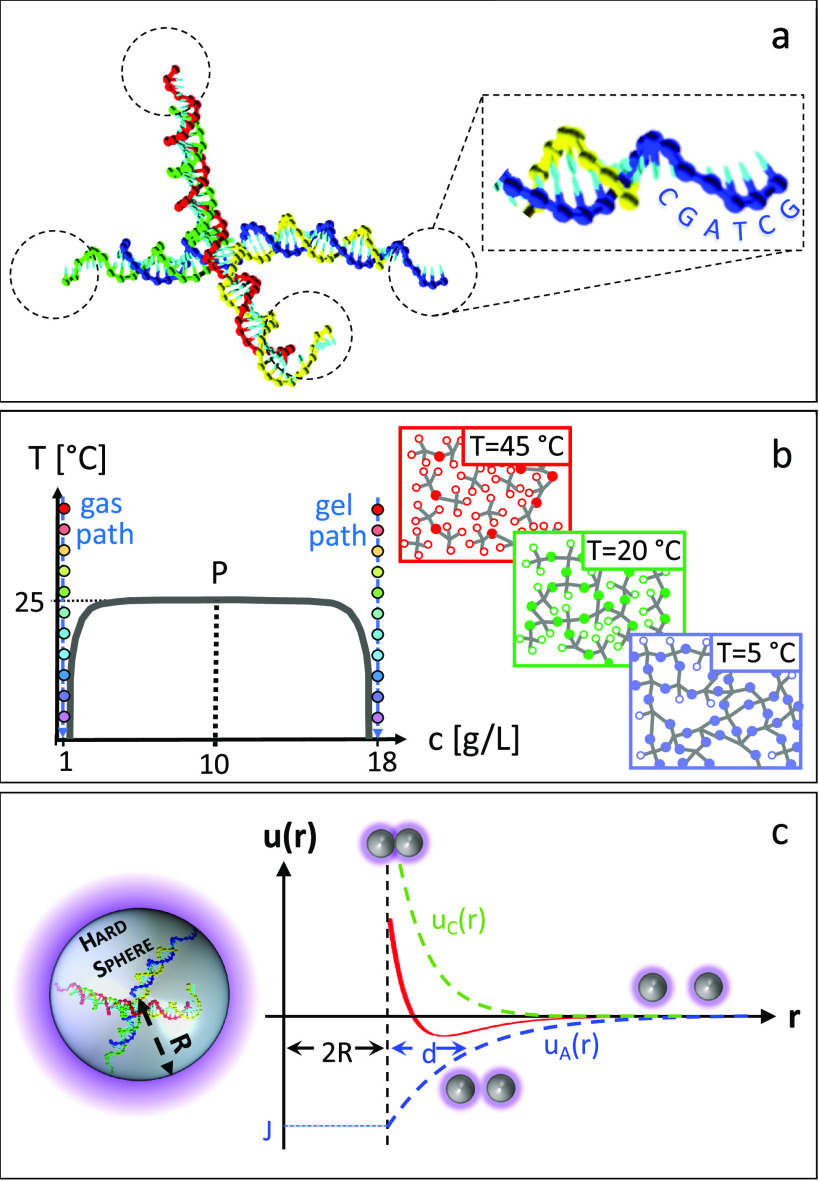
Sketch of the main features of the investigated DNA nanostar system. (a) DNA nanostars are formed by the aggregation of four distinct strands into a structure with fours double strands connected at the center, terminating with a six-base-long overhang carrying identical palindromic sequences (CGATCG). The hybridization of the overhangs provides the *T*-dependent mechanism of aggregation. (b) The phase behavior of solutions of DNA nanostars exhibits a vapor–liquid-type coexistence curve. Here we study two solutions whose concentrations are at the left (“gas path” at 1 g/L) and at the right of the coexistence curve (“gel path” at 18 g/L). In both conditions the systems remain in a single phase at all *T*. (c) The interaction between DNA nanostars is modeled as an isotropic pair potential *u*(*r*) (red curve) resulting from the combination of a hard-sphere core of radius *R*, a screened electrostatic repulsive potential *u*_C_(*r*) (green dashed line), and an attractive potential *u*_*A*_(*r*) (blue dashed line) of depth *J* and range *d*.

DNA-made nanostars constitute a interesting class of DNA nanoconstructs for which functionality (or valence) and binding strength can be independently controlled. These particles are multistranded self-assembled structures shaped as sketched in Figure 1a. Each “arm” of the nanostar—in the number of four in this study—terminates with a short overhang whose sequence is palindromic. The hybridization of the overhangs, occurring at a temperature *T* that depends on their length and sequence, leads to tip-to-tip adhesion, in turn yielding the formation of clusters and eventually of spanning networks. These structures have found important applications as model systems to investigate colloidal gelation^7^ (both chemical^8,9^ and physical^10−12^ gelation), network viscoelasticity,^13−16^ liquid–liquid phase transitions,^17,18^ and the dependence of the phase-behavior on the valence.^10^ These particles have also been used as elementary bricks in more complex architectures to give rise to solutions gelling on heating^19^ or to assemble cold-swappable networks.^20^

Because of the strong *T* dependence of the overhang hybridization free energy, the phase diagram of DNA nanostars features—within a range of only 20 °C—a coexistence curve ending in a critical point and a path for reversible gelation,^10−12^ as sketched in Figure 1b. Despite their wide applicability, detailed information on the evolution of the microscopic structure during the aggregation of DNA-nanostars is still missing. Light scattering,^10−12^ microscopy, and rheological experiments^13−16^ have characterized the macroscopic response, both in terms of time dependence of the density fluctuation on a spatial scale comparable to the wavelength of visible light and in terms of frequency dependence of the elastic moduli (again in the bulk limit). The emerging picture is that of a reversible network, whose restructuring follows the opening and closing of the interparticle bonds, with a bond lifetime which varies by more than 4 orders of magnitude on changing temperature.

The experiments and analysis reported here focus on solutions of DNA nanostars at two concentrations flanking the coexistence curve, as sketched in Figure 1b. In both cases, the systems remain homogeneous at all *T*. Along the gas path at low concentration, the system always remains in the vapor-like phase, while in the gel path (the liquid-like side of the coexistence curve) the system undergoes gelation without incurring phase separations. Indeed, previous studies^11,12^ have shown that the kinetic arrest of the system along the gel path takes place through equilibrium states, with the characteristic time depending only on *T* and the light scattered intensity leveling off at the lowest investigated *T*, suggesting that at those *T* a fully bonded network is formed.

While neutron and X-rays scattering experiments are the ideal candidates for exploring the structure and dynamics of DNA-based structures,^21,22^ DNA nanostars have been so far explored only via small-angle neutron scattering experiments in dilute conditions.^23^ Here we report synchrotron small angle X-ray scattering (SAXS) experiments on solutions of tetravalent DNA nanostars along the gas and the gel paths. We aim at determining the structural evolution of the system along the two paths, extracting an effective nanostar–nanostar potential and its *T* concentration and dependence.

To this aim we devised a data analysis strategy comprising several steps. The form factor of the DNA nanostar has been evaluated taking advantage of equilibrated molecular dynamics (MD) trajectories with a new method (sasDNA) that takes into account the scattering contribution of the hydration water. The structure factor has been calculated by adopting a spherically averaged effective potential, obtained by the sum of a hard-sphere core, a repulsive screened Coulomb term, and an attractive Yukawa term (Figure 1c), and by using the perturbative random phase approximation of the Percus–Yevick equation.^24^ By means of a fitting procedure we extracted the values and *T*-dependence of the effective hard-sphere radius *R*, and of the depth *J* and width *d* of the Yukawa attractive potential. We find that despite the simplifying assumptions, our theory describes well the SAXS observations. The Yukawa parameters that we extract from the gas and gel path SAXS data are basically identical, confirming the effectiveness of our simple description. We find a mild *T* variation, with the attractive Yukawa potential becoming a bit stronger and wider when the nanostar tips hybridize, as it could be expected. We also find that the nanostar concentration affects the value and *T* dependence of *R*. The effective hard core appears generally larger along the gel path, possibly reflecting the different conformations accessible to the nanostars at the different concentrations.

## Materials and Methods

### Sample Preparation

To assemble DNA particles with limited valence *f* = 4 we dissolved equimolar quantities of four distinct 49-mers single-strand (ss) DNA. The 49-mers sequence is composed by three sections, two of which of length 20 bases and one (so-called) palindromic terminal sequence of length six bases (CGATCG). To enable angular flexibility among different arms, bases with no complementary partner were inserted in between these three section. The two sections of length 20 bases are complementary to other sections on distinct strands providing, on binding, the self-assembly of the tetramer arms (See Figure S1 in the Supporting Information (SI)). Since the length of the overhangs—6 bases—is much smaller than the arms—20 bases—their melting temperature *T*_*b*_ ≈ 25 °C is well separated by the temperature at which the 20 bases melt *T* ≈ 65 °C. Therefore, a large *T* interval exists in which constructs are well formed but weakly interacting. To perform SAXS experiments, we prepared dilute (*c* = 1 g/L, *I*_*S*_ = 74 mM) and dense (*c* = 18 g/L, *I*_*S*_ = 22 mM) solutions of DNA nanostars (see Sample Preparation section in the SI). Such concentrations were chosen so to always remain in the homogeneous regions of the phase diagram, as sketched in Figure 1b.

### Simulated DNA Nanostar Configurations

To evaluate the form factor of tetravalent DNA nanostars, we simulated a nanostar with the OxDNA code.^25^ The OxDNA interaction potential, parametrized against experimental results, reproduces structural and thermodynamic properties of both single- and double-stranded molecules of DNA in B-form. Nucleotides are modeled as rigid bodies. The interactions between nucleotides account for excluded volume, backbone connectivity, Watson–Crick hydrogen bonding, stacking, cross-stacking, and coaxial-stacking. From the molecular dynamics trajectory, *N* = 29 independent configurations (coordinates and orientations of all 196 nucleotides) have been saved on disk and used to evaluate the form factor as described in the following.

### SAXS

#### Experimental Setup

SAXS data were collected at the highflux Austrian beamline at Elettra Synchrotron in Trieste, Italy.^26^ Measurements were carried out in the same capillaries where samples were prepared (1.00 mm outer diameter, 0.01 mm wall thickness), enclosed within a thermostatic compartment connected to an external circulation bath and a thermal probe for temperature control. A heating and a subsequent cooling temperature ramp was set between 5 and 45 °C, with steps of 5 °C from 5 to 20 °C and from 30 and 45 °C and steps of 2 °C between 20 and 30 °C. Two dimensional patterns were recorded by a Pilatus3 1 M detector system, data were stored in TIF format and then processed with FIT2D^27^ to apply a mask for beamstop and detector and to perform the radial average of isotropic signals. Considering that the photon flux density at the capillary was ≈4 × 10^11^ s^–1^, we measured each sample 23 times with an acquisition time of 20 s and a rest time of 500 s for each temperature step. Raw data were calibrated in absolute units (cm^–1^) using water. The sample-to-detector distance was set to 1.74 m, which provided *q*, the modulus of the scattering vector **q** according to *q* = 4π sin θ/λ, 2θ being the scattering angle and λ = 1.54 Å being the X-ray wavelength, equal to 0.014–0.3 Å^–1^. Tetravalent DNA nanostar solutions and their buffers were measured at the same conditions concerning temperature and exposure time. The scattering curves have been normalized to the primary beam intensity, corrected for sample transmission, and normalized to absolute scattering units. We did not observe radiation damage on samples presented in this study.

#### SAXS Model

The experimental observable obtained by the SAXS technique, called the “macroscopic differential scattering cross section”, is expressed by the following equation
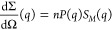
1where *n* is the number density of the particles in solution, *P*(*q*), the mean orientational averages of the excess scattering amplitude and of its squared modulus (this latter referred to as the “form factor”), and *S*_*M*_(*q*), the “measured structure factor”, a term that depends on the average radial correlation between the positions of all the DNA nanostars. Considering that in-solution DNA nanostars occupy a volume fraction lower than ≈0.02 and that water density changes in the investigated temperature range for ≈1% of its value, it has been necessary to evaluate the dependence of *n* on the absolute temperature *T*. *n*(*T*) has been calculated on the basis of the bulk water relative mass density, *d*_w_(*T*), with respect to the reference temperature *T*_o_ = 298.15 K, according to *n*(*T*) = *cN*_A_*d*_w_(*T*)/*M*_DNA_, where *c* is the nominal weight/volume concentration of DNA nanostars at *T*_o_, *M*_DNA_ is the DNA nanostar’s molecular weight, and *N*_A_ is Avogadro’s number. We have approximated the data of *d*_w_(*T*), derived by the results of Kell,^28^ in our investigated range 5–45 °C, by the following function of *T*
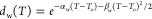
2by finding the optimum value of the thermal expansivity at *T*_o_, α_w_ = 2.5 × 10^–4^ K^–1^, and its first derivative, β_w_ = 9.8 × 10^–6^ K^–2^.

#### Form Factor

The form factor developed for this study describes the average structure of randomly oriented DNA nanostars, which we build on the basis of their coarse grained model as obtained by molecular dynamic simulations with the OxDNA code. It has been calculated via an original method that exploits the multipole expansion of the Debye law, on the basis of equilibrium MD configurations of DNA nanostars described by a coarse grained model. Each *m* molecule is described by the position vectors **r**_*k*,*m*_ of all the effective spheres representing its *k* united-atom, classified, according to the primary sequence, in five possible groups (labeled with *j*): 1, the DNA backbone (phosphate and deoxyribose), 2, adenine, 3, guanine, 4, cytosine, and 5, thymine (the four DNA bases). The X-ray excess scattering amplitude of the *m* molecule is written as a combination of the united-atoms in the water term (at) and the first hydration shell term (sh), as in standard methods such as CRYSOL^29^ or SASMOL:^30^
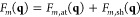
3The united-atoms in the water term is
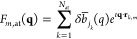
4where the sum runs over all the *N*_at_ united atoms of the DNA nanostar and involves the excess scattering length of the *j*th of five groups, , approximated by the square root of the orientational average of the squared excess scattering amplitude due to all the *a* atoms belonging to the group, this latter calculated with the Debye law
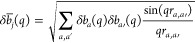
5In this equation, *r*_*a*,*a*′_ is the distance between the *a* atom and the *a*′ atom constituting the group (we have considered standard values obtained by crystal structures of DNA molecules), and *δb*_*a*_(*q*) is the difference between the scattering length of the *a* atom in vacuum and the scattering length of the water displaced by that atom
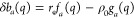
6where *r*_e_ = 0.28 × 10^–12^ cm is the classical radius of the electron,  is the Thomson excess scattering amplitude (the Fourier transform of the atomic electron density, assumed to be spherically symmetric, approximated by a combination of five Gaussians as reported by Waasmaier and Kirfel^31^), ρ_0_ is the scattering length density (SLD) of the bulk water (calculated for X-rays as ρ_0_ = *r*_e_*n*_e,w_ρ_*m*,w_^°^*N*_A_*d*_w_(*T*)/*M*_w_, *n*_e,w_ = 10 being the number of electrons of the water molecule, ρ_*m*,w_^°^ the water mass density at *T*_o_, and *M*_w_ the water molecular mass) and *g*_*a*_(*q*) is the Fourier transform of a spherical Gaussian that represents the distribution of a portion of water that occupies the van der Waals volume ν_*a*_ of the *a* atom
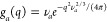
7The number *N*_*h*_ and the centers **r**_*k*,*m*_ of the dummy spheres in contact with the united atoms of the DNA nanostar, representing first hydration water molecules, are found according to the SASMOL method,^30^ and their excess scattering amplitude contribution can be written as
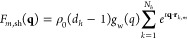
8where *d*_*h*_ is the relative mass density of the hydration water with respect to the bulk water and the spherical Gaussian *g*_w_(*q*) is calculated considering the molecular volume ν_w_ of bulk water molecules. To notice, both amplitudes *F*_*m*,at_(**q**) and *F*_*m*,sh_(**q**) are subsequently expanded in a series of spherical harmonics, in order to easily calculate the orientational averages (i.e., the averages over the polar angles α_*q*_ and β_*q*_ of **q**) of the DNA nanostar amplitude, , and its square modulus,  (see the work of Ortore et al.^30^ for further details). Considering all the *N* molecules of an equilibrated MD trajectory, it is straightforward to calculate mean values of both terms
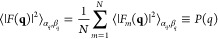
9
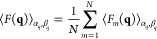
10where the mean squared term in eq 9 is finally the form factor, *P*(*q*), of the DNA nanostar. This model, called sasDNA, has been now implemented in the freely available software GENFIT.^32^

#### Structure Factor

The measured structure factor is defined by
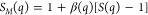
11where *S*(*q*) is the particle–particle structure factor and β(*q*) is the coupling function
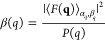
12which could significantly deviate from 1 for anisotropically shaped molecules,^33,34^ such as DNA nanostars. We assume, to calculate *S*(*q*), that the radial interaction potential *u*(*r*) between two molecules is described by the hard sphere double Yukawa (HSDY) model, which is constituted by a hard sphere (HS) term, a screened Coulomb term (C) and an attractive term (A), according to
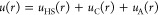
13
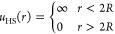
14
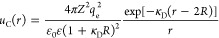
15
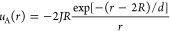
16The physical parameters contained in these equations are *R*, the apparent, effective radius of the particle; *Z*, the net number of elementary electric charges, *q*_e_ = 1.6 × 10^–19^ C, the proton charge in SI units; ε_0_, the vacuum permittivity; ε, the relative dielectric constant, which has been approximated by that of pure water, whose dependency on temperature is known;^35^, the reciprocal Debye–Hückel screening length, which depends on the ionic strength I due to all *i* microions, , (*I*′ is the DNA nanostars’ counterions contribution, *I*_*S*_ is the one of the added salts), *z*_*i*_ being the charge number, *C*_*i*_ the molar concentration, *k*_B_ Boltzmann’s constant; *J*, the depth of the attractive potential at contact (*r* = 2*R*); *d*, the characteristic length scale of the attractive potential, outside the hard sphere. Considering volume fractions of DNA nanostars in our samples lower than ≈0.02, the particle–particle structure factor, *S*(*q*), has been expressed as a perturbation of the Percus–Yevick structure factor, *S*_0_(*q*), in the framework of the random-phase approximation (RPA),^24,34,36^ according to
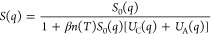
17
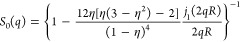
18where  is the DNA nanostar volume fraction,  is the analytic isotropic Fourier Transform of the Yukawa potential *u*_*j*_(*r*) = *B*_*j*_ exp[−κ_*j*_(*r* – 2*R*)]/*r*, with *j* = C and A, and *j*_1_(*x*) is the first spherical Bessel function.

The treatment of the parameters that describe the radial particle–particle interaction deserves a word of caution. (i) Considering that at neutral pH the phosphate group of each DNA basis is completely deprotonated, the absolute value of the charge number *Z* of DNA nanostars should be fixed to the total number of residues, which is 196 for our molecule. (ii) Assuming monovalent counterions (*z*′ = 1), on the basis of the electroneutrality principle, the molar concentration of counterions, which determines the contribution *I*′ to the total ionic strength *I*, can be easily determined according to *C*′ = |*Z*|*cd*_w_(*T*)/*M*_DNA_, and hence considered a known property of the sample as a function of *T*. (iii) The effective radius of the DNA nanostars cannot be considered constant, since, on one hand, they are flexible molecules and, on the other, their average dimension may depend on the structure of the phase they form (dense or dilute) and, more in general, on the temperature. (iv) A careful evaluation merits the parameters *J* and *d* describing the attractive term merit a careful evaluation. Indeed, differently from the screening Coulomb potential, which is based on the consolidated Debye–Hükel theory, the choice of a similar Yukawa form for the attractive potential is not based on theoretical arguments but only on the ease calculation affort of its Fourier transform. What is certain is that the formation of a network of DNA nanostars is due to the Watson–Crick base pairing between the complementary six-nucleotide-long overhang of sequence CGATCG, which is clearly an anisotropic attraction.

Hence, the attractive Yukawa potential we have adopted is a simple approximation, in isotropic radial symmetry, of the directional attraction between DNA nanostars, and within this approximation, it is wise to consider that both parameters *J* and *d* may depend on either the phase formed by DNA nanostars and the temperature.

#### Global Fit

It is known that, in general, the information content of a single SAXS curve is low and that, in order to derive in a robust way the physical parameters associated with a system, it is better to analyze, with a unique model, a batch of SAXS curves obtained on the same system by varying one or several chemical–physical conditions.^32,37^ In this framework, we distinguish between the fitting parameters that are common to all the curves and the ones that refer to a single curve. In our model the single curve parameters are the average radius *R*, the depth *J*, and the range *d* of the attractive potential. Another strategy that, on one hand, increases the robustness of SAXS data analysis and allows small modifications of the physical parameters and, on the other, avoids unlikely oscillations of them for close values of chemical–physical conditions, is to adopt a regularization algorithm.^38−40^ According to these requirements, all the SAXS curves, recorded at the different temperatures belonging to a continuous ramp, for a fixed concentration of DNA nanostars in water (dense or dilute), have been globally analyzed by minimizing the following merit function
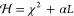
19The first term, χ^2^, is the average standard reduced chi-square of the *m*th experimental curve, , over *N*_*m*_ experimental curves
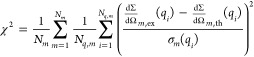
20where *N*_*q*,*m*_ is the number of *q* points of the *m* curve, σ_*m*_(*q*_*i*_) is the experimental standard deviation, and  is the theoretical curve calculated on the basis of eq 1. The second term, *L*, is the regularization factor
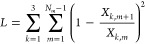
21which increases with the difference of the values of the *k*th single curve fitting parameter, *X*_*k*,*m*_ (*k* = 1, 2, 3 refers to *R*, *J*, and *d*, respectively), of two close conditions, corresponding to the *m* curve and the (*m* + 1) curve. The constant α in eq 19 is carefully chosen to guarantee that, for values of χ^2^ close to 1 (indicating a good global-fit), the factor *αL* is lower than ≈10% of whole merit function . The relative mass density of the hydration water with respect to the bulk water, *d*_*h*_, has been considered a smooth function of temperature, according to , where the two parameters *d*_*h*_^°^ (the value at *T*_o_) and α_*h*_ (the associated thermal expansivity) are treated as common parameters of all the SAXS curves of a batch.

## Results and Discussion

**Figure 2:**
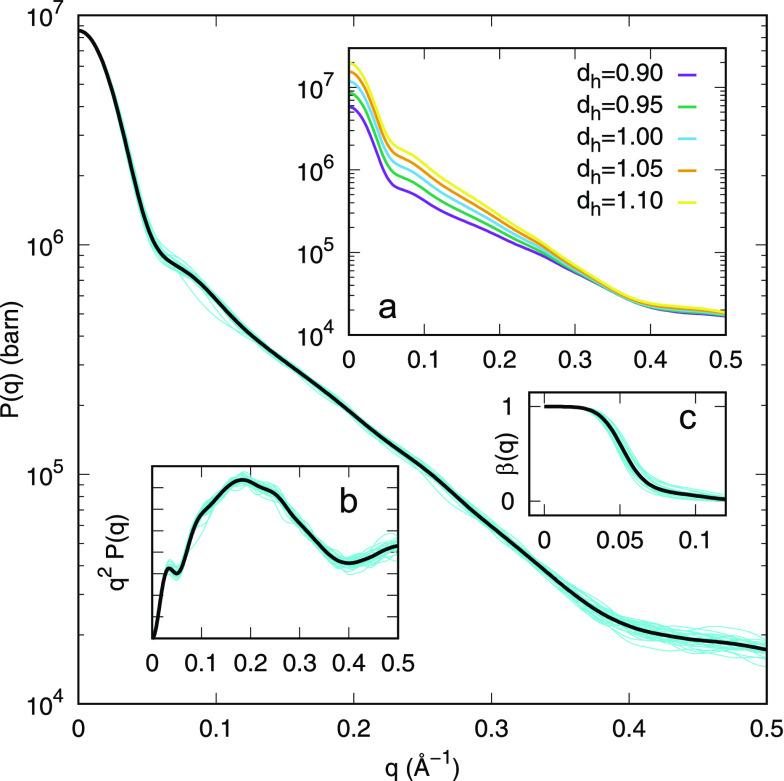
Form factors of *N* = 29 tetrameric MD DNA nanostar structures (cyan curves, simulated at 70 mM NaCl and 30 °C) and their mean value (black curve). (inset a) Mean values of *P*(*q*) for different relative mass densities *d*_*h*_ of the hydration shell. (inset b) Kratky plots of the curves reported in the main panel. (inset c) Coupling functions corresponding to the curves reported in the main panel.

Form factors of *N* = 29 tetrameric DNA nanostar structures, obtained with the MD method at 70 mM NaCl and 30 °C, calculated with eq 9, are shown, in a semilog graph, in the main panel of Figure 2 (cyan curves), together with their mean value (the black curve). The peculiar behavior of *P*(*q*), which does not resemble the one of simple geometrical shapes, reflects the structural characteristics of a four-arms, partially flexible, particle. In the inset a of Figure 2, we report the mean values of *P*(*q*), calculated for different relative mass densities of the hydration shell, *d*_*h*_: the marked variations are due to a large number of hydration molecules that encompass the four arms of the DNA nanostar molecule. The inset b of Figure 2 shows the same data of the main panel in form of Kratky plot: the presence of a dominant peak centered at *q* ≈ 0.2 Å^–1^ indicates that the structure of the molecules is quite compact. The behavior of the coupling function β(*q*) (inset c of Figure 2) is calculated from eq 12 and clearly shows that β(*q*) value rapidly (at *q* ≈ 0.1 Å^–1^) falls to zero.

**Figure 3:**
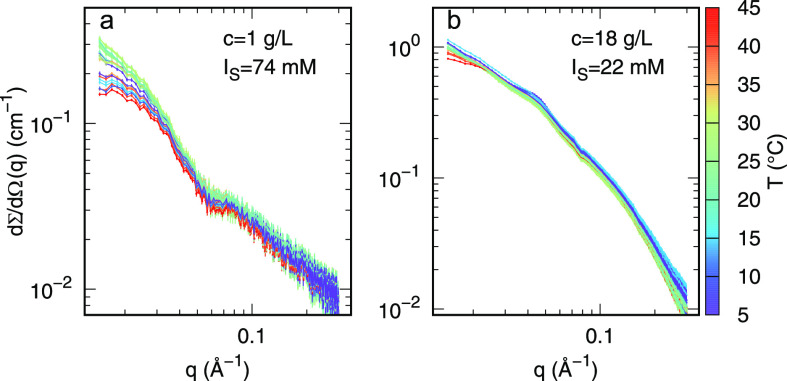
Experimental SAXS curves of dilute (a) and dense (b) DNA nanostar samples, recorded at different temperatures within the heating and cooling ramps, according to the values shown in the color box.

Experimental synchrotron SAXS curves, for dilute (*c* = 1 g/L) and dense (*c* = 18 g/L) water solutions of DNA nanostars, are reported in Figure 3 (panels a and b, respectively), as a function of *T*. SAXS experimental points of the same color correspond to samples measured in heating and cooling ramps, matching the same temperature. Note that the overlapping of SAXS data of the same color is easily appreciated, confirming the reversibility of the process. The two sets of SAXS experiments (Figure 3) appear quite different: a marked variation with temperature at low *q* occurs for the dilute sample (panel a), while this change is less evident for the dense sample (panel b). Only the SAXS curve corresponding to the dilute sample at the highest temperature shows an asymptotic behavior at low *q* resembling a Guinier trend, suggesting the absence of significant correlation among the particles at higher temperatures. All SAXS data of the dense sample (panel b) show the presence of a small bump at *q* ≈ 0.04 Å^–1^, almost independent of *T*. This feature, absent in the form factor (Figure 2), indicates a more structured organization of the particles’ network.

These preliminary observations of SAXS data have lead us to work out the model described in the previous section, which may be suitable for an in-deep interpretation of the whole set of SAXS experiments, by taking into account both effects due to the form factor and to the structure factor of DNA nanostars.

**Figure 4:**
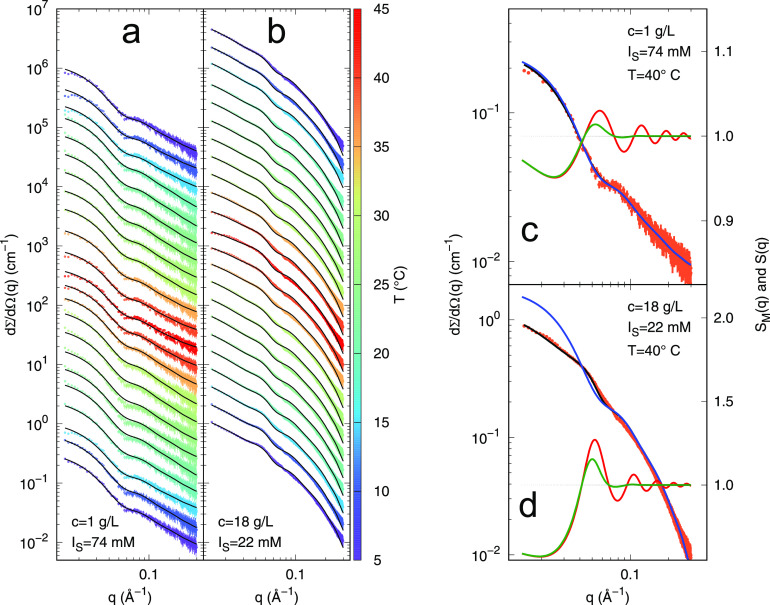
Experimental SAXS curves of dilute (a) and dense (b) DNA nanostar samples, recorded at different temperatures within the heating and cooling ramps, according to the values shown in the color box. Black solid lines are the best fit obtained by two global-fit analyses. From the bottom to the top, curves are shown following the temperature ramp and subsequently multiplied by a factor 2, for clarity. (c and d) SAXS experimental data corresponding to *T* = 40 °C with the best fit curve (black line) and the *n P*(*q*) curve (blue line) are shown; the measured structure factor *S*_*M*_(*q*) (green line) and the particle–particle structure factor *S*(*q*) (red line) are referred to the right *y*-axis.

We performed two independent global-fit analyses: one for SAXS curves of the dilute sample and another for the ones of the dense sample. Corresponding best fit curves are reported as solid black lines in Figure 4 (panel a and b, respectively). For each series, the two common parameters of the set are the relative mass density of the hydration water at *T*_o_, *d*_*h*_^°^, and its thermal expansivity α_*h*_. The parameters related to each curve and constrained by the regularization procedure (eq 21) are the average radius *R* of the DNA nanostar, the depth *J* and the range *d* of the attractive potential. For dilute and dense samples, the merit function , defined in eq 19, results 0.80 and 2.34, respectively, whereas the corresponding values of χ^2^ are 0.70 and 2.25. The relative mass density of the hydration shell, *d*_*h*_^°^, results 1.001 ± 0.008 at *c* = 1 g/L and 1.0505 ± 0.0003 at *c* = 18 g/L, with negligible variation with *T*, being, for both cases, α_*h*_ < 10^–5^ K^–1^.

**Figure 5:**
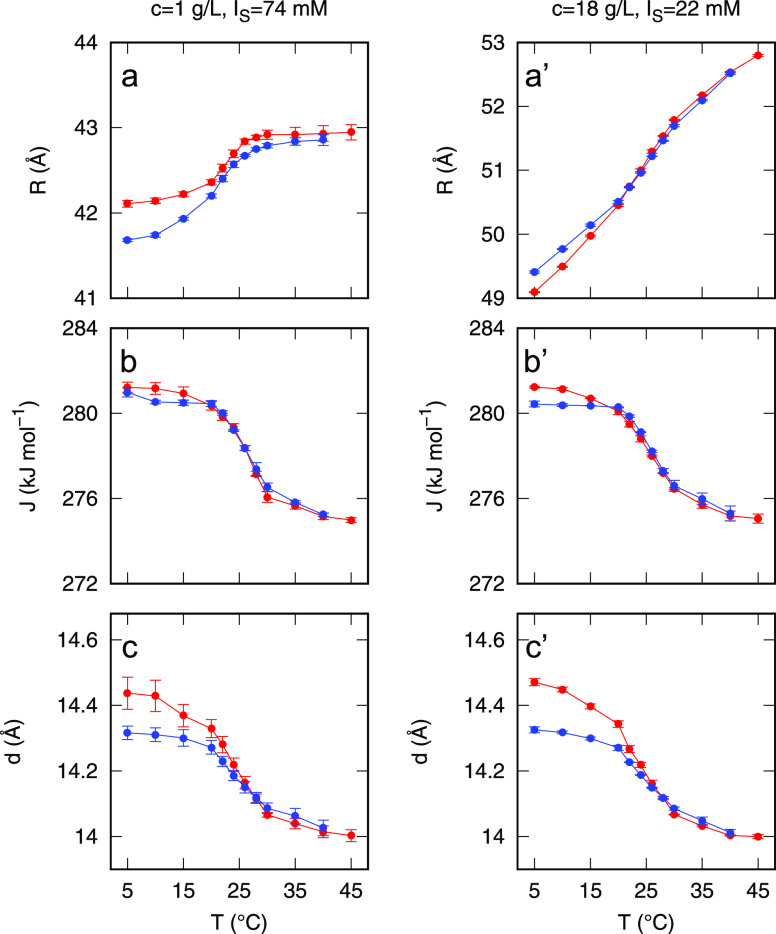
Temperature dependence of the average radius *R* (a and a′), the attractive potential energy at contact *J* (b and b′), and of the range of the attractive potential *d* (c and c′) for the dilute DNA nanostar sample (left column, unprimed panels) and the dense sample (right column, primed panels). Red and blue symbols refers to the heating and the cooling portion of the temperature ramp, respectively.

The interaction parameters *R*, *J*, and *d* resulting from the global fitting procedures are reported as a function of *T* in Figure 5. Parameters obtained by fitting the SAXS curves measured upon heating are represented in red, whereas those obtained from the cooling are shown in blue. In all cases, there is a good superposition between heating and cooling trends of the parameters, confirming the expected reversibility of the process. The residual difference between heating and cooling data at low *T* is probably indicating that the equilibration time adopted in the experiments was not sufficient in that regime, in which the sample kinetics is particularly slow. The variation of the parameters in the explored *T* range is small, in the order of a few percent. This variation is however significant in the fitting process since, if these parameters are constrained to fixed values, the χ^2^ values becomes much larger. All three parameters, for both dilute and dense samples, follow a sigmoidal curve centered around *T* ≈ 25 °C, corresponding to the critical temperature of the phase separation for tetrameric DNA nanostars (Figure 1b, adapted from the work of Biffi et al.^10^).

The apparent radius *R* of DNA nanostars is ≈42 Å (Figure 5a) for the dilute sample, a figure close to the hydrodynamic radius *R*_*h*_ ≈ 47 Å, measured by dynamic light scattering at large *T* in a 10 g/L sample.^10^*R* is instead larger in the dense sample, ranging from 49 to 53 Å (Figure 5a′).

At low *T*, the large density sample is almost fully bonded. The internanostar bond involves the hybridization of the 6-base-long overhangs, which thus convert from the highly flexible DNA single strand to rigid DNA double strands of approximate length of 20 Å, to be split between the two interacting particles. This picture should be compared with the range *d* of the Yukawa attractive interaction, which we find to be rather large, ≈14 Å. While this finding could appear in contradiction with the precise positioning of the overhangs required to form a bond, we instead argue that it could be explained considering the wide conformational space intrinsic to the nanostars, in which the arms are free to rotate and the unpaired bases in the central junction enable breathing. Indeed, the length of fully extended arms and open junction—a condition probably very rare—is about 90 Å.

The values of the energy at contact, *J*, are in the range 276–280 kJ/mol. When compared with the enthalpy associated with the hybridization of each overhang (≈160 kJ/mol),^10^ the values that we obtain for *J* suggest that each pair of nanostars binds to each other through ≈1.5 bonds.

As *T* increases, all parameters change. The change is centered around ≈25 °C, the *T* at which the overhangs melt and the nanostars unbind. At high *T* the nanostars are on average further away from each other and their transient interactions take place at the distance at which the arm terminals first come into contact. We can thus understand the growth of *R* with *T* as an effect of the large conformational space, which is thermally explored on time scales shorter than those relative to center-of-mass diffusion across a particle radius, so that the effective hard sphere radius increases with *T*. This effect appears more relevant for the sample at larger concentration where the low *T* state is of full bonding.

The small decrease of *J* and *d* with *T* is instead harder to understand. We argue it reflects the transient nature of the bonds at high *T*, by which do not form for long time enough to completely stabilize, thus resulting in a partial exploration of the potential well.

It is worthy of notice that the values of *J* and *d* are basically identical in the dilute (Figure 5b) and dense samples (Figure 5b′). This indicates not only that the assumption of spherical effective interaction is actually working well and does not require adjustment when the sample concentration is modified, but that is able to capture the key energy terms involved in the binding of the flexible arms.

**Figure 6:**
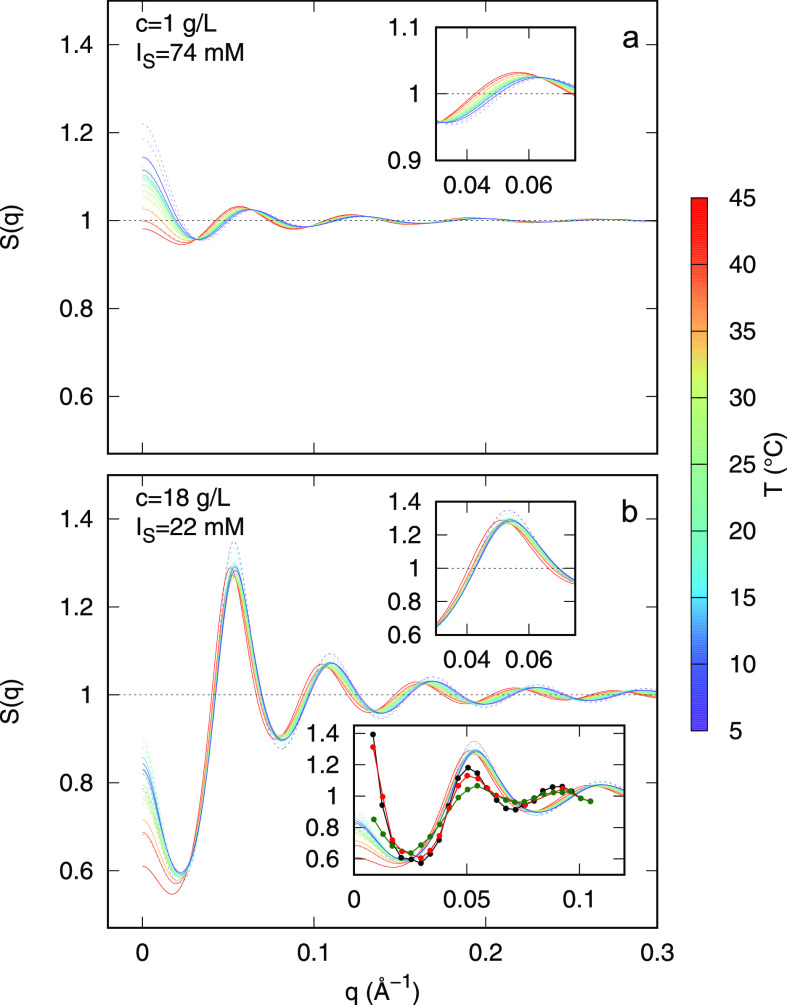
Particle–particle structure factors of dilute (a) and dense (b) DNA nanostar samples obtained by the analysis of SAXS data. Curves are color-coded according to the temperature values reported in the color box. Solid and dashed lines refer to the heating and cooling portions of the ramp, respectively. The upper insets of each panel report an enlargement of the first peak of the structure factor. The lower inset in panel b shows a comparison between the structure factor resulting from SAXS data at *c* = 18 g/L and the one arising from numerical simulations at *c* = 24 g/L, temperature of 39 °C (black), 42 °C (red), and 45 °C (green).^41^

The particle–particle structure factors *S*(*q*) obtained by the two global-fit analyses are reported in Figure 6a, for the dilute sample, and in Figure 6b, for the dense sample. Curves have been calculated in a *q*-range larger than the experimental one (from *q* = 0) to compare these results with theoretical calculations (lower inset in panel b of Figure 6). In the dilute case, *S*(*q*) slightly oscillates around 1 and, at low *q*, tends toward 1 at the largest temperatures, as expected because the progressive absence of correlation among the particles. As detailed in the inset of panel a, the first peak slightly shifts toward lower *q* by increasing *T*. This nearest-neighbor peak of the *S*(*q*) is related to the intertetramer average distance, and in the real space distance, it moves from ≈100 Å at 5 °C to ≈112 Å at 45 °C. Note that the estimated value for two bonded tetramers in the minimum of the energy is ≈148 Å.^41^

For the dense sample the *S*(*q*) (Figure 6 b) is much more structured and shows changes as a function of *T* at low *q*. The first peak position (emphasized in the inset of panel b) slightly moves toward lower values of *q* by increasing temperature. The change of the first peak position of the *S*(*q*) with temperature for the dilute sample and the concentrated sample are noticeably different, reflecting the different influence of *T*. In both cases, however, the overall change in *S*(*q*) with *T* is weak. This is particularly interesting in the case of the dense system which, upon cooling, undergoes a gel transition. Our results indicate that such kinetic arrest occurs with almost no structural effect. This result strengthens the peculiarity of gels formed by DNA nanostars, that not only transform from thin fluids into viscoelastic medium in an interval of few degrees, but do so without undergoing local rearrangements and, hence, without producing local stresses. Thus, these gels, perfectly biocompatible, could be considered as promising materials to realize matrices holding delicate microscopic biological structures.

The general behaviors of the resulting *S*(*q*) are in agreement with the theoretical ones^41^ as shown in the lowest inset of Figure 6b: the first peak position falls in the region close to *q* ≈ 0.05 Å^–1^, and mostly for the dense sample, its height decreases by increasing *T*. Moreover, data of Rovigatti et al.^41^ for dense samples indicate that, by increasing *T*, *S*(*q*) at small *q* strongly decreases, reaching the value *S*(*q*) ≈ 0.7 at *T* = 48 °C, in good agreement with our result for *c* = 18 g/L at *T* = 45 °C (Figure 6, panel b, red curve).

Although this study is based on just two different concentrations of DNA nanostars, it is useful to note that the first peak position of *S*(*q*) for *q* ≈ 0.05 Å^–1^ (Figure 6 top insets) is essentially independent of the DNA nanostar concentration, as also observed in the numerical simulations of Rovigatti et al.^41^

The whole detailed library of SAXS curves at each temperature, together with their best fit, their corresponding graphs of *S*_*M*_(*q*) and *S*(*q*) and the model parameters, are reported in Figures S2 and S3 in the SI. To note, the effect of the coupling function β(*q*), as reported in Figure 2, inset c, provokes a marked difference between *S*_*M*_(*q*) and *S*(*q*): it is evident that only the first peak of the *S*(*q*) affects the fitting curve, whereas the other peaks are mostly damped by β(*q*). This effect is quite general when scattering particles have a marked shape anisotropy and merits to be underlined because it is not universally focused on by the SAXS community. In this regard, it is worth underlining that the HSDY-RPA model here applied is just one possible simple approximation, in radial symmetry, of a more complex anisotropic structure factor. Other approximated models of *S*(*q*), once damped by the effect of β(*q*), may equally well fit the data.

**Figure 7:**
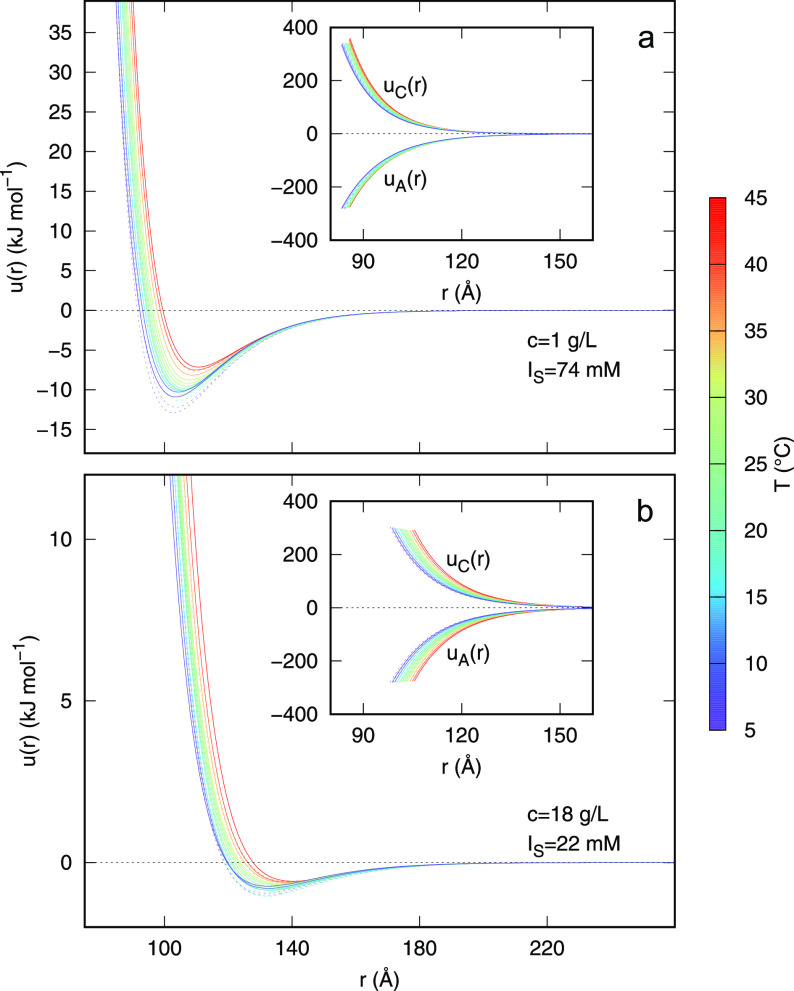
Pair interaction potentials of dilute (a) and dense (b) DNA nanostar samples obtained by the analysis of SAXS data. The repulsive and attractive terms are reported in the insets. Curves are color-coded according to the temperature values reported in the color box. Solid and dashed lines refer to heating and cooling portions of the ramp, respectively.

Another way to explore the obtained results of the present SAXS analysis is to consider the variations of the potential energy *u*(*r*) with *T* and concentration. To note, *u*(*r*) is the orientational and conformational average of the anisometric pair potential *u*_12_(**r**_12_, Ω_1_, Ω_2_, Φ_1_, Φ_2_), which depends on the vector distance **r**_12_ between the centers of two DNA nanostars and, more importantly, on both their orientation, in general described by two set of Euler angles Ω_1_ and Ω_2_, and their conformation, described by the two sets Φ_1_ and Φ_2_ of the necessary dihedral angles. As a matter of fact, *u*_12_(**r**_12_, Ω_1_, Ω_2_, Φ_1_, Φ_2_) must be independent of *T* and *c*, whereas its approximated average, *u*(*r*), could change with the systems conditions. Plots of the SAXS derived *u*(*r*) are reported in Figure 7, panel a, for the dilute sample, and in Figure 7, panel b, for the dense sample. In the insets of both panels, we report the behavior of the screened Coulomb term *u*_C_(*r*) (curves in the positive region) and of the attractive term *u*_A_(*r*) (curves in the negative region). It is worth noticing that for both dilute and dense samples the repulsive and the attractive terms, which are in the order of 300 kJ/mol at low distance, almost compensate each other, giving rise to a depth of the minimum of the *u*(*r*) of approximately 10 and 1 kJ/mol at 1 and 18 g/L, respectively. This rather counterintuitive result could be explained considering that the difference in the depth can be only attributed to the difference in the average radius, being all the other parameters defining *u*(*r*) almost the same. Indeed, at the shorter distances *r* that can be reached in the dilute case, both attractive and repulsive Yukawa functions (eqs 15–16) show a more marked slope so that, when they are added, a deeper minimum arises. We also notice that by increasing *T*, the depth decreases and the position of the minimum moves to higher distance, suggesting a more disordered system.

## Conclusion

Shape and interactions of DNA nanostars in solution are intertwined in the set of SAXS data here presented. By taking advantage of numerical simulations, we have developed a fairly accurate model for the form factor, and we have disentangled the information concerning the interactions from the ones concerning the molecular shape. We have shown that this method (sasDNA) can calculate the form factor of DNA nanostars considering MD equilibrated trajectories and by taking into account the hydration shell contribution, too. Noticeably, the star-like shape of the particles leads to a coupling factor that strongly dampens the modulations of the structure factor. The coupling between the sasDNA method and the determination of the structure factor in the framework of the simple HSDY model for describing the pair potential between two DNA nanostars allows to fit in a accurate way the whole set of SAXS curves for the dilute sample as well as the dense sample.

The approach adopted here involves the dramatic simplification of the actual anisotropic nanostar interaction into a spherically symmetric potential. While it is known that a single-minimum isotropic potential resulting from a short-range repulsion term (*u*_HS_(*r*) + *u*_C_(*r*) in the present case) complemented by a attraction contribution (*u*_A_(*r*)) cannot predict that shape and location (in density) of the gas–liquid coexistence,^42^ we find that the dual approach here adopted (i.e., interpreting SAXS data via an isotropic potential) is very effective in gaining structural information.

Our analysis leads to the first experimental determination of the structure factor and its dependency on concentration and temperature for a DNA nanostars system. *S*(*q*) profiles derived from the SAXS curves are in good agreement with the theoretical calculation obtained by means of molecular dynamics simulations.^41,43^ The values of the parameters used to fit the data are all in agreement with expectations, confirming the effectiveness of the method. Moreover, the (mild) temperature dependence of these parameters enlightens subtle features, which could emerge only through a detailed structural analysis, as the one here presented. We find that the effective radius of the DNA nanostars increases with both concentration and temperature, while the attractive component of the interaction is identical at the two concentrations while decreasing with temperature. This behavior is all very reasonable and can be accounted for by expected features of the system. In particular, the strength attractive interaction indicates that, at full bonding, each pair of DNA nanostars forms on average, 1.5 bonds, a previously unrecognized feature of the system which is, again, quite reasonable. We also find that the strong electrostatic repulsion between DNA nanostars is nearly compensated by the attraction forces arising from the Watson–Crick pairing of arms.

## Supporting Information Available

Sample preparation and Figures S1–3 (PDF)
