# Supplementary material for: Gelling without Structuring: A SAXS Study of the Interactions among DNA Nanostars
Source: Langmuir. 2020 Aug 10;36(35):10387–96. doi: 10.1021/acs.langmuir.0c01520 (PMC8010795; doi:10.1021/acs.langmuir.0c01520)
Supplement: Supplementary file 1 — la0c01520_si_001.pdf [file la0c01520_si_001.pdf]

Supporting Information

Gelling without structuring: a SAXS study of the interactions among DNA nanostars

Francesco Spinozzi[1*], Maria Grazia Ortore[1], Giovanni Nava[2], Francesca Bomboi[3], Federica Carducci[1], Heinz Amenitsch[4], Tommaso Bellini[2*], Francesco Sciortino[3], Paolo Mariani[1]

[1] *Department of Life and Environmental Sciences, Polytechnic University of Marche, Ancona, Italy*

[2] *Department of Medical Biotechnology and Translational Medicine, Università degli Studi di Milano, Milan, Italy*

[3] *Department of Physics, Sapienza, Università di Roma, Rome, Italy*

[4] *Institute for Inorganic Chemistry, Graz University of Technology, Graz, Austria*

* E-mail: f.spinozzi@univpm.it * E-mail: tommaso.bellini@unimi.it

Sample preparation

The DNA nanostar results from the self-assembly of four 49-nucleotide-long base sequences (See Fig. S1).

The preparation and the characterization of the DNA nanostars solution was done according to Biffi et al.[1]. In detail, each sequence was hydrated using a 22 mM NaCl solution. In this way, assuming that each phosphate group dissociates a cation, we fixed the total ionic strength of 76 mM. Each of the four sequence solutions was then carefully analysed by measuring the absorbance at 260 nm (Thermofisher Nanodrop) in order to obtain the same molarity.

The four sequence solutions were then mixed in the same eppendorf obtaining a nanostar solution of 10 g/L; the concentration used in the main text for SAXS measurement (18 g/L) was in fact too viscous to be handled properly. To ensure an optimal hybridization of the DNA sequences the sample was annealed in a thermocycler by increasing the temperature to 90° C and slowly cooling down to room temperature (approx. 3 hours).

The sample was then put in a glass capillary (3 mm OD) and centrifuged at 10° C for 12 hours until a clear phase separation is observed[1]. The dense phase at the bottom of the capillary (18 g/L "dense" sample) was then put in thin quartz capillaries (Hampton Research, 1 mm OD, 0.98 ID).

A part of the initial sample at 10 g/L was diluted at 1 g/L and the NaCl concentration was

adjusted to 74 mM in order to match the total ionic strength of the dense 18 g/L sample. The "dilute" sample was then put in the quartz capillaries.

Each capillary was sealed with glue to avoid evaporation during SAXS measurements.

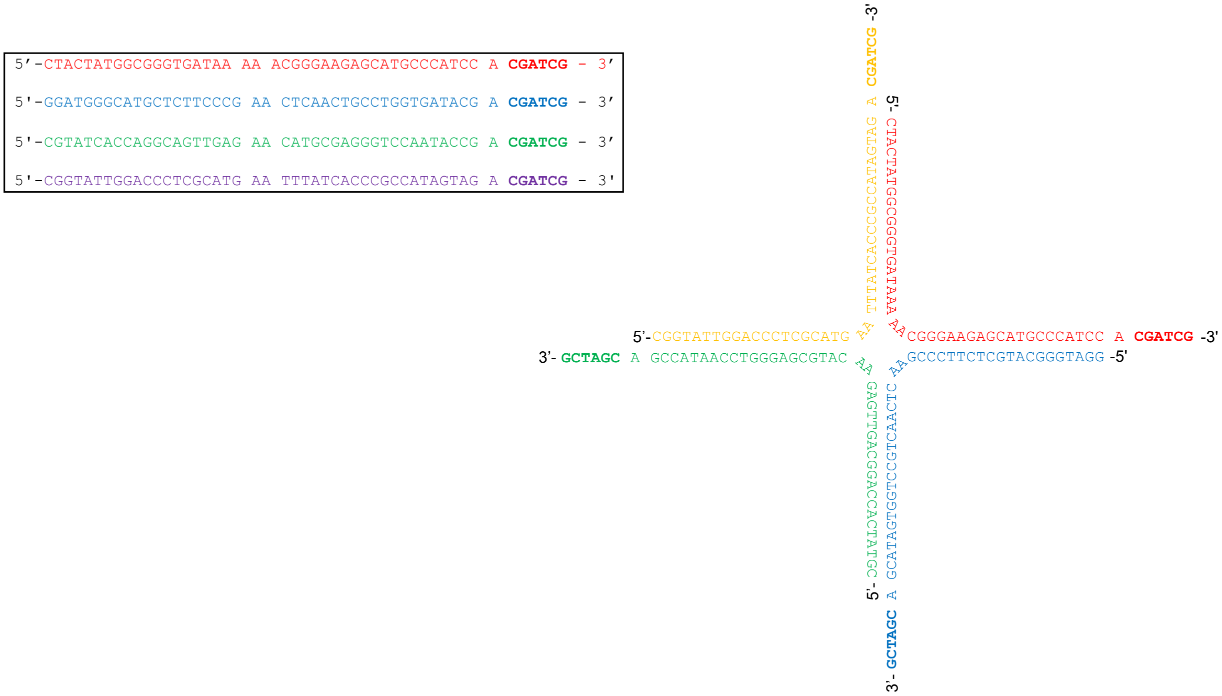

Figure S1: The DNA nanostars are built from the self-assemble of 4 DNA strands. The sequences were designed so that the final structure is formed by 4 double strand arms with the same free palindromic sequence of 6 bases at each tip (CGATCG).

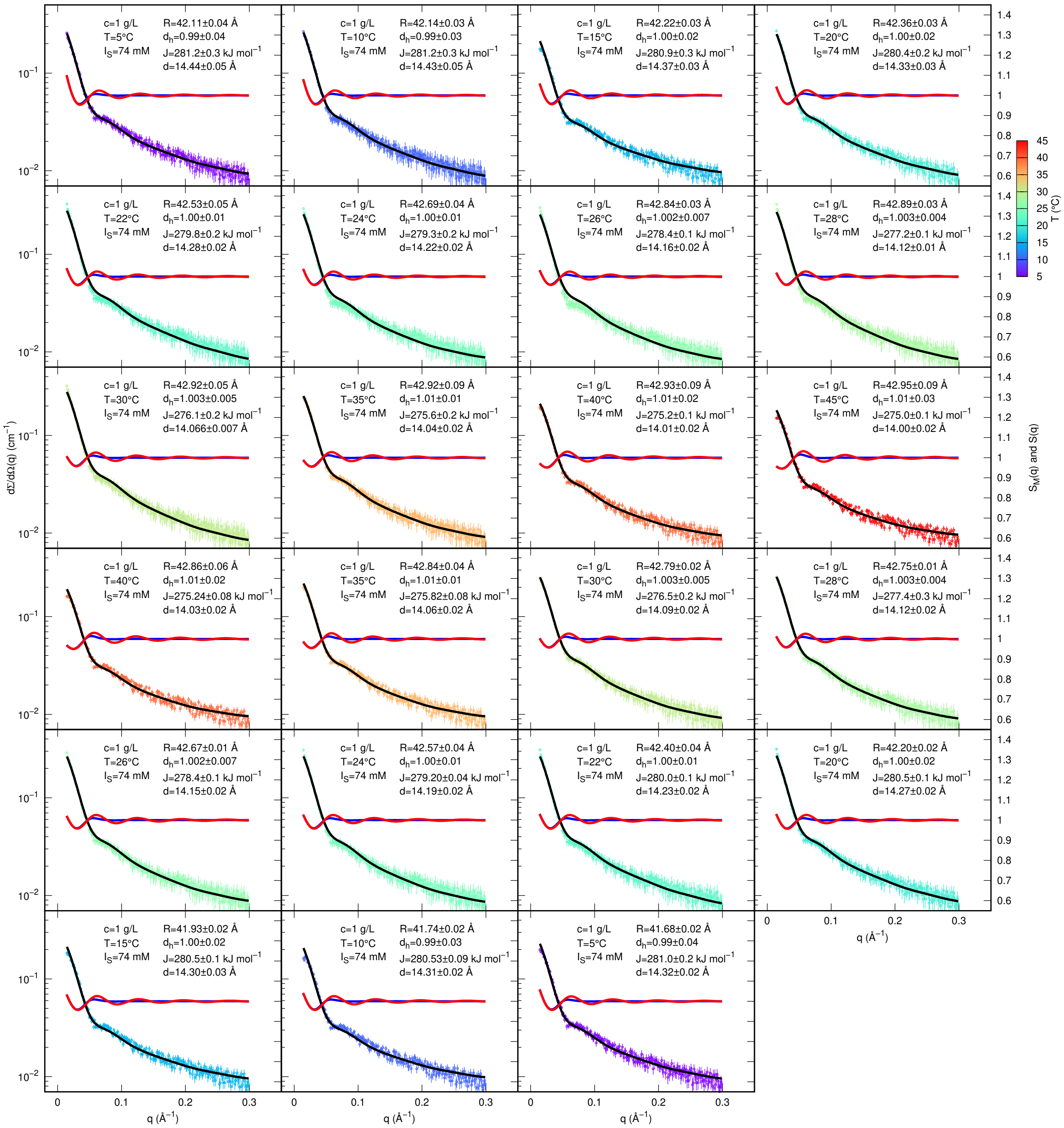

Figure S2: Detailed plots of the analysis of the SAXS curves recorded at different temperatures on heating and on cooling of the diluted DNA nanostar sample. Each panel reports: the experimental curve (points with error bars, color-coded according to the color box); the best fit curve (solid black line); the measured structure factor $S_M(q)$ and the particle-particle structure factor $S(q)$ (blue and red solid lines, respectively, referred to the right y-axis); the main fitting parameters.

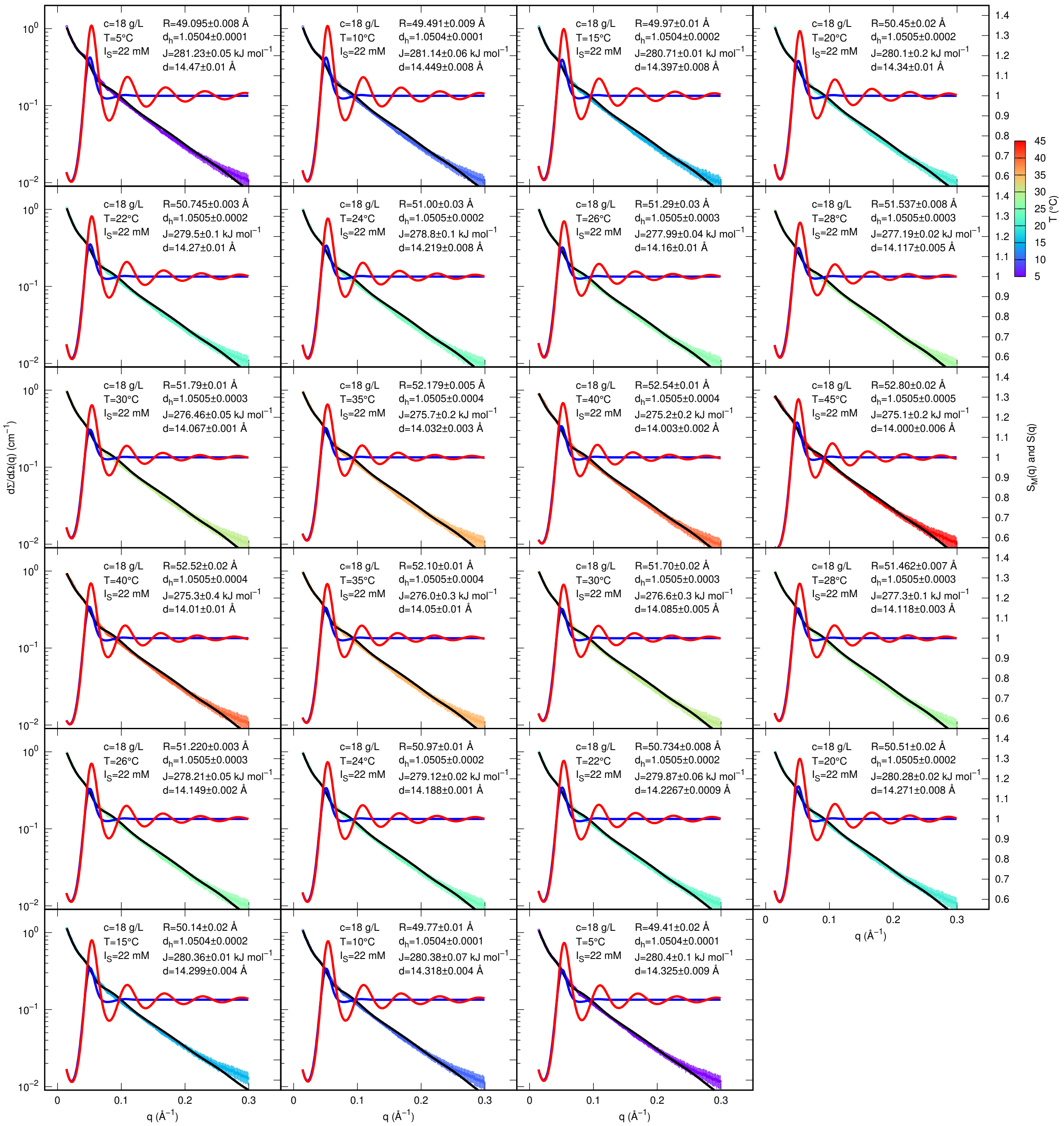

Figure S3: Detailed plots of the analysis of the SAXS curves recorded at different temperatures on heating and on cooling of the dense DNA nanostar sample. See the caption of Fig. S2 for details.

References

[1] Biffi, S.; Cerbino, R.; Bomboi, F.; Paraboschi, E. M.; Asselta, R.; Sciortino, F.; Bellini, T. Phase behavior and critical activated dynamics of limited-valence DNA nanostars. *Proceeding of the National Academy of Sciences* **2013**, *110*, 15633–15637.
